# Cooling effect of evaporative misters in outdoor restaurant areas: case study of Novi Sad, Serbia

**DOI:** 10.1007/s00484-026-03203-7

**Published:** 2026-04-24

**Authors:** J. Dunjić, V. Květoňová, S. Manavvi, D. Milošević, S. Savić, V. Stojanović, M. Lehnert

**Affiliations:** 1 Department of Geography, Tourism and Hotel Management, Faculty of Science, University of Novi Sad, Novi Sad, Serbia; 2 Department of Geography, Faculty of Science, Palacký University Olomouc, Olomouc, Czech Republic; 3 Department of Complex Systems, Institute of Computer Science of the Czech Academy of Sciences, Prague, Czech Republic; 4 Department of Architecture and Planning, Indian Institute of Technology, Roorkee, India; 5 Meteorology and Air Quality Section & Hydrology and Environmental Hydraulics Section, Wageningen University & Research, Wageningen, Netherlands

## Abstract

Continuing urbanization, together with climate change manifestations, including the increasing frequency and intensity of heat waves, prompts the adoption of various cooling interventions in urban outdoor spaces. Evaporative misting systems have gained popularity as a cost-effective heat mitigation measure, particularly in hospitality venues. However, their effectiveness in moderate climates remains understudied. This study evaluated the effectiveness of these systems in restaurant outdoor seating areas in Novi Sad, Serbia, during hot summer days in 2022 and 2023. Simultaneous micrometeorological measurements were conducted at multiple locations in restaurant outdoor seating areas (sun-exposed, shaded, misted, and non-misted sites), with mean radiant temperature (MRT), Physiological Equivalent Temperature (PET), and Universal Thermal Climate Index (UTCI) being calculated. Additionally, to capture subjective perceptions of microclimatic conditions, questionnaire surveys were conducted with restaurant guests. Results revealed significant location-specific variability in cooling effectiveness. The most favorable thermal conditions occurred in shade; however, in sun-exposed locations, misting systems showed inconsistent performance, sometimes even worsening thermal conditions. Despite these inconsistencies, questionnaire results showed that guests consistently perceived improved thermal comfort under misting systems. Further research should include measurements across different times of day and seasons in moderate climates and across different misting types and intervals to better understand misting systems’ cooling benefits.

**Supplementary Information:**

The online version contains supplementary material available at 10.1007/s00484-026-03203-7.

## Introduction

Climate change and intensive urbanization affect not only citizens’ outdoor thermal comfort, but also overall living conditions and health. It is reported that extreme heat conditions, such as heat waves, are expected to further increase in intensity and frequency in Europe (Fischer and Schär 2010; Jacob et al. 2018). This means that during hot days, especially during heat waves, normal outdoor activities may be jeopardized. Additionally, during these days, local businesses that depend on outdoor spaces, such as restaurants and cafes with outdoor seating, are affected too.

In recent decades, many interventions that aim to mitigate the effects of heat stress have emerged. They can be classified into three categories: gray (e.g. shading options, cool roofs, etc.); vegetation-based – green (e.g. urban parks, street trees, etc.); and water-based – blue interventions (water bodies, misting systems, etc.) (Huang et al. 2025). Some interventions are rather passive and have limited controllability options (Montazeri et al. 2017). Other mitigating strategies, such as evaporative cooling misters, are applicable in various areas and allow more flexibility and dynamics in control (Wang et al. 2023).

Cooling misting systems as a heat mitigation measure in the built environment have gained popularity recently for urban public, residential, commercial, and industrial applications, due to their low cost, localized cooling effect, and easy control options (Meng et al. 2022). Evaporative misting systems usually include a water source, a pump, a long and flexible pipeline, and nozzles (Huang et al. 2025). The nozzles convert the water into a highly pressurized mist of fine water droplets (Vanos et al. 2022). Studies by Zhang et al. (2021) and Oh et al. (2019) show it is a well-accepted heat mitigation technique in practice.

Over the past two decades, numerous studies have assessed evaporative misting for thermal comfort, with outcomes varying by climate. According to available literature, these systems work best in hot and arid climates and/or in subhumid conditions (Esparza-López et al. 2022; Dhariwal et al. 2019; Vanos et al. 2022), while in more humid (sub)tropical climates (Huang et al. 2025; Oh et al. 2019; Xie et al. 2024; Wang et al. 2024; Zhang et al. 2021) and temperate climates (see below), these systems show lower effectiveness in temperature reduction and thermal comfort improvement.

Evaporative misting systems are also popular in urban areas in European climates. For example, in Italian cities with a Mediterranean climate, studies by (Ulpiani, 2019; Ulpiani et al., 2019) and Di Giuseppe (2021) analyzed air temperature and relative humidity in an urban park and found that cooling misters reduced the air temperature by 8 °C but increased relative humidity by 7%. On the other hand, in the temperate climate of Czechia, Lehnert et al. (2021a) conducted field measurements in Pilsen, and showed that misting systems have a low impact on the reduction of heat stress (on average 0.4 °C UTCI). When combined with (tree) shade, the results are better (up to 8.5 °C UTCI).

Although evaporative misting systems can be used in a variety of settings, they are often implemented in hospitality facilities such as restaurants, bars, and cafes, to attract customers (Vít and Kopp 2019). However, this implementation often occurs without well-grounded guidelines that elaborate on conditions for their maximum cooling potential, considering local conditions and climatic contexts (Ulpiani et al., 2019). Most studies on misting systems rely on experimental setups, with few measuring their effectiveness in real-world conditions (e.g. Vanos et al. 2022; Xie et al. 2024). Beyond measurements, examining the perceptions of users is also beneficial to gain a comprehensive understanding of the general usefulness of misting systems in open areas during hot summer days. Therefore, the main objective of this study is to empirically examine the effectiveness of cooling misting systems in open spaces associated with restaurants and cafes in a central European city in terms of thermal exposure as well as human thermal sensation. This objective will be achieved through these specific objectives:

i) To estimate the outdoor thermal comfort using two biometeorological indices (PET and UTCI) and mean radiant temperature (MRT) during hot summer days in restaurants with outdoor seating capacity equipped with evaporative misting systems.
ii) To examine restaurant guests’ perceptions of misting systems and their effectiveness in improving thermal sensation.

With this approach, we aim to address the following research questions:

### RQ1

How do evaporative misting systems modify microclimatic parameters (air temperature, relative humidity, wind speed, and globe temperature) in outdoor hospitality spaces in temperate continental climate conditions?

### RQ2

How do these microclimatic modifications influence thermal comfort indices (PET and UTCI) in sun-exposed and shaded restaurant environments?

### RQ3

What are the practical implications of these findings for the design and operation of misting systems in private outdoor hospitality spaces?

## Study area and methods

### Study area

Novi Sad is the second-largest city in Serbia, located in the southern part of the Pannonian Basin (80–86 m a.s.l.), on the northern bank of the Danube River (45°15′N, 19°50′E). Two water bodies flow through the city: the Danube River and the Danube-Tisa-Danube Canal. South of the Danube lie the slopes (90–200 m a.s.l.) of the low-lying Fruška Gora Mountain (Milošević et al. 2022a). Novi Sad has a Cfb temperate climate (Kottek et al. 2006), with the coldest month being January with a mean of -0.3 °C and the warmest being July with a mean of 21.8 °C. The mean annual precipitation is 623 mm for the period 1949–2015 (Savić et al. 2018). The urbanized area covers 112 km^2^ with a population of 325,000 (Milošević et al. 2022a) and is characterized by a compact historic center surrounded by modern residential neighborhoods.

The field studies were conducted at seven restaurants with outdoor seating areas equipped with evaporative misting systems, during the summers of 2022 and 2023. The restaurants are located across various urban morphologies in the city of Novi Sad, differing in surface cover, vegetation, and green infrastructure, which influence their microclimatic conditions. To provide a general idea about their surroundings, we added information about the Local Climate Zones (LCZ) of each restaurant. LCZ for Novi Sad were determined by Savić et al. (2018). The locations of each measurement site (restaurant) are presented in Table 1, and 2. Detailed characteristics of the sites are provided in the supplementary material (Tab. S1).

**Table 1:**
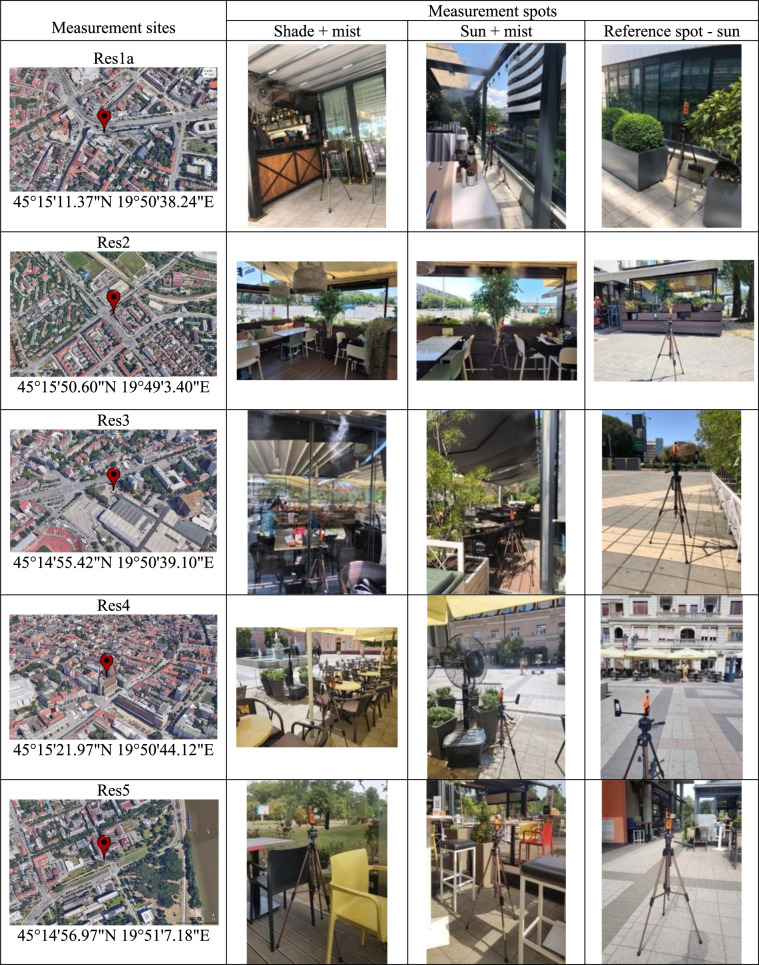
Measurement sites in Novi Sad in the summer of 2022

**Table 2:**
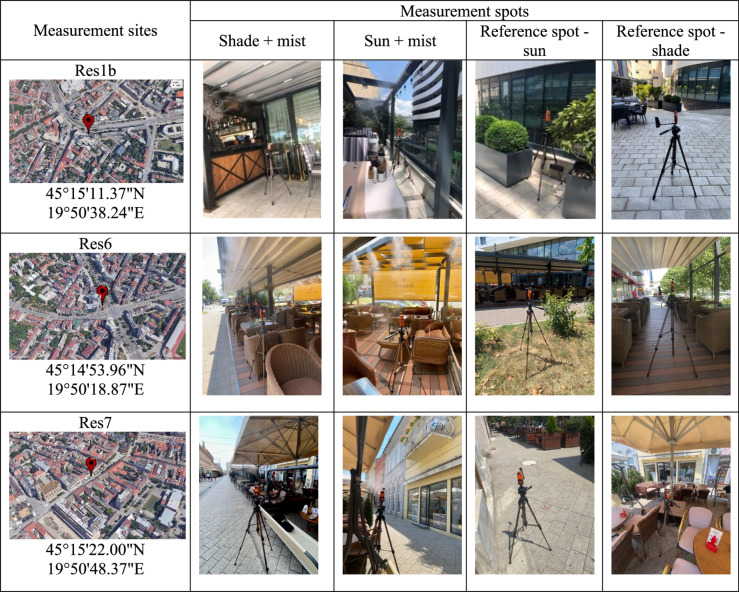
Measurement sites in Novi Sad in the summer of 2023

#### Study sites characteristics

Based on records provided by a local misting systems installation company (https://www.te-cooling.rs/reference/ugostiteljski-objekti), 39 hospitality establishments in Novi Sad were identified as having installed evaporative misting systems. Through additional field verification, 7 further establishments were identified, resulting in at least 46 misting-equipped venues in the city at the time of the measurement campaigns. Seven restaurants were included in these measurement campaigns, which represents approximately 15% of the identified hospitality establishments using misting systems. All investigated misting systems operated in intermittent on–off cycles, with “on” phases of approximately 10–20 s and “off” phases of 10–30 s (see Tab. S1), and the duration of both phases was systematically recorded on-site during the measurement campaigns. The restaurant locations have distinct urban morphological characteristics and solar exposure patterns. The measurements were conducted during days with comparable meteorological conditions in both campaigns. Each restaurant was designated as “*ResX*” in chronological sequence to preserve anonymity. Two measurement campaigns were conducted. The first took place in summer 2022 at five restaurants; the second was conducted in summer 2023 at three restaurants. Measurements were taken at three to four spots per restaurant: a sun-exposed spot away from the misters’ reach (“sun” as a reference spot), a sun-exposed spot with mist (“sun+mist”), a spot in shade with mist (“shade+mist”), and a spot in shade away from the misters’ reach (“shade”). The “shade” spot was added in the 2023 campaign. Based on the lessons learned from the 2022 campaign, in 2023 we adjusted the measurement timeframe from 2 to 5 p.m. to 1–4 p.m.

### 2022 campaign

The first restaurant (“Res1a”) is situated in a street with a northeast orientation, within the high-rise building complex, directly overlooking Narodnih Heroja Street. The location experiences sun exposure during morning hours, while midday and afternoon periods are shaded by tall surrounding buildings. This site was remeasured in 2023 (“Res1b”). The second restaurant (“Res2”) occupies an open midrise setting with a northeast orientation, directly facing Rumenačka Street. This site receives sun exposure during morning and midday hours, with afternoon hours becoming partially shaded by adjacent buildings and trees. The third restaurant (“Res3”) is located in a compact low-rise environment with a northeast orientation, directly facing Sutjeska Street. The site experiences sun exposure during morning and midday hours, with afternoon shading provided by trees and nearby buildings. The fourth restaurant (“Res4”) features a compact low-rise setting with northwest orientation, directly facing the square in front of the Church of the Name of Mary. During morning hours, the site is partially shaded by nearby buildings and the church, while midday and afternoon periods experience full sun exposure. The fifth restaurant (“Res5”) represents an open midrise configuration with southwest orientation towards a green space. The site experiences partial shading from buildings and trees during morning hours, with midday and afternoon periods characterized by direct sun exposure.

### 2023 campaign

Measurements in 2023 were conducted at three locations. The first measurement site was the same as in 2022, Res1b (see above). The second restaurant (“Res6”) is positioned in an open street near a major crossroad with a southwest orientation, directly facing Braće Ribnikar Street. The location experiences partial shading during morning hours, with midday and afternoon periods characterized by direct sun exposure. The last restaurant (“Res7”) is located in the city center on an old town street with a southeast orientation, directly facing Zmaj Jovina Street. This site receives sun exposure during morning and midday hours, with afternoon hours becoming partially shaded by nearby buildings.

### Measurements

#### Instrument parameters

We used three (2022 campaign) and four (2023 campaign) mobile Kestrel 5400 Heat Stress Trackers to measure air temperature (T_*a*_ in ℃), relative humidity (RH in %), wind speed (v in m s^− 1^), and globe temperature (T_*g*_ in ℃) with one-minute resolution. The measurements were performed at 1.1 m above ground, representing the approximate center of gravity of standing subjects (ISO 7726 1998). The Kestrel Heat Stress Trackers werea deployed at least 10 min before the start of each measurement session to allow the sensors to adjust to the local atmospheric conditions at the site. The instruments were calibrated according to the manufacturer’s specifications, and their accuracy values comply with ISO 7726 (1998) standards for sensor measurement range and accuracy (Tab. S2). Similar measurement settings were already successfully applied in previous studies (Milošević et al. 2022b, c).

#### Measurement protocol 1 – summer 2022 

We conducted 3-hour measurements (from 2 p.m. to 5 p.m.) using three Kestrel devices placed on outdoor terraces equipped with misting systems at five restaurants. The devices were placed simultaneously at three spots in each restaurant: “sun”, “sun+mist”, and “shade+mist” (see Sect. 2.1.1. Study sites characteristics).

Measurements were conducted at each establishment on the following days: Monday, July 4, Thursday, August 18, and Friday, August 19. Calm, clear, and hot days were selected for measurements (see Tab. S3 for background weather data), as previous studies (e.g., Ulpiani et al., 2019) reported that the spray cooling effect was more apparent and easily measurable in still air conditions.

#### Measurement protocol 2 – summer 2023

 We conducted 3-hour measurements (from 1 p.m. to 4 p.m.) using four Kestrels placed on outdoor terraces at three restaurants with misting systems. The devices were placed simultaneously at four spots in each restaurant: “sun”, “sun+mist”, “shade”, and “shade+mist” (see 2.1.1. Study sites characteristics). Measurements were conducted on the following days: Saturday, July 15, Sunday, July 16, and Monday, July 17, under calm, clear, and hot conditions (Tab. S3).

The measurement campaigns in 2022 and 2023 were conducted on different days due to instrument availability constraints. Consequently, synoptic conditions and, in the case of 2022, solar position were not identical across all measurement days. However, measurements were performed during stable summer high-temperature conditions, and comparable meteorological contexts were selected to ensure consistency of analysis.

### Calculation of indices

For the bioclimatic analysis and outdoor thermal comfort assessment, Mean Radiant Temperature (MRT), Physiologically Equivalent Temperature (PET), and Universal Thermal Climate Index (UTCI) were selected, with PET and UTCI being the most frequently applied indices in outdoor thermal comfort research (Potchter et al. 2018). Measured meteorological parameters (T_a_, RH, T_g_, and v) were averaged over 10-minute periods for the calculation of bioclimatic indices. MRT was calculated using the formula from ISO 7726 (1998):

$$\:MRT={\left[{(Tg+273.15)}^{4\:}+\frac{hcg}{\epsilon\:\:\cdot\:\:{D}^{\mathrm{0.4}}\:}\cdot\:(Tg-Ta)\right]}^{\mathrm{0.25}}\:-\:\mathrm{273.15}$$

where T_*a*_, T_*g*_, and *v* were obtained from in situ measurements, D represents globe diameter (25 mm), ɛ represents globe emissivity, and hcg represents globe’s mean convection coefficient, (hcg = 1.1 ∙ 10^8^ ∙ Va^0.6^, Kántor and Unger, 2011).

Using the calculated MRT and measured values for T_*a*_, RH, and *v*, along with default values for personal characteristics (age, height, weight, clothing, and metabolic rate), PET and UTCI values were calculated for each measurement site. PET was calculated using the RayMan model (Matzarakis et al. 2007), while UTCI was calculated using the official UTCI calculator (www.utci.org). PET and UTCI categories and index thresholds according to Błażejczyk et al. (2014), Jendritzky et al. (1990), and Matzarakis et al. (1999) are provided in Tab. S4.

For clarity, results of MRT, PET, and UTCI are presented as differences compared to the sun-only site, as exemplified here with MRT:

$$\triangle MRT = MRT \scriptsize x \normalsize - MRT \scriptsize sun-only$$

where MRT_*x*_ represents the value at each listed site (sun+mist, shade, or shade+mist (2023 only)). This was done similarly with PET and UTCI.

### SHAP-based sensitivity analysis

A SHAP-based sensitivity analysis was conducted to quantify the relative importance of four micrometeorological parameters—air temperature (T_a_), globe temperature (T_g_), relative humidity (RH), and wind speed (v)—in determining PET, UTCI, and MRT under four microclimatic conditions: sun, shade, shade+mist, and sun+mist. The mean absolute SHAP value for each parameter was calculated across the sampled data, representing the average magnitude of each parameter’s impact and providing a measure of relative importance.

### Questionnaire survey

To complement the quantitative measurements and calculated indices, a questionnaire survey was conducted (see Fig. S1). It targeted restaurant guests to assess how misting systems affected their subjective thermal sensation and comfort perceptions. The questionnaire survey was designed to be concise and consisted of five short questions, to avoid prolonged disturbance of the guests. The questionnaire included two questions about personal information (gender and age) and three questions about respondents’ current thermal sensation and their perception of the effectiveness of the misting systems. Guests were surveyed during the measurement periods, after being briefly informed about the study purpose. The relatively small number of respondents (*n* = 86) reflects the limited restaurant occupancy during the extreme weather conditions on measurement days.

## Results

### Measurement results

#### Summer 2022

**Fig. 1:**
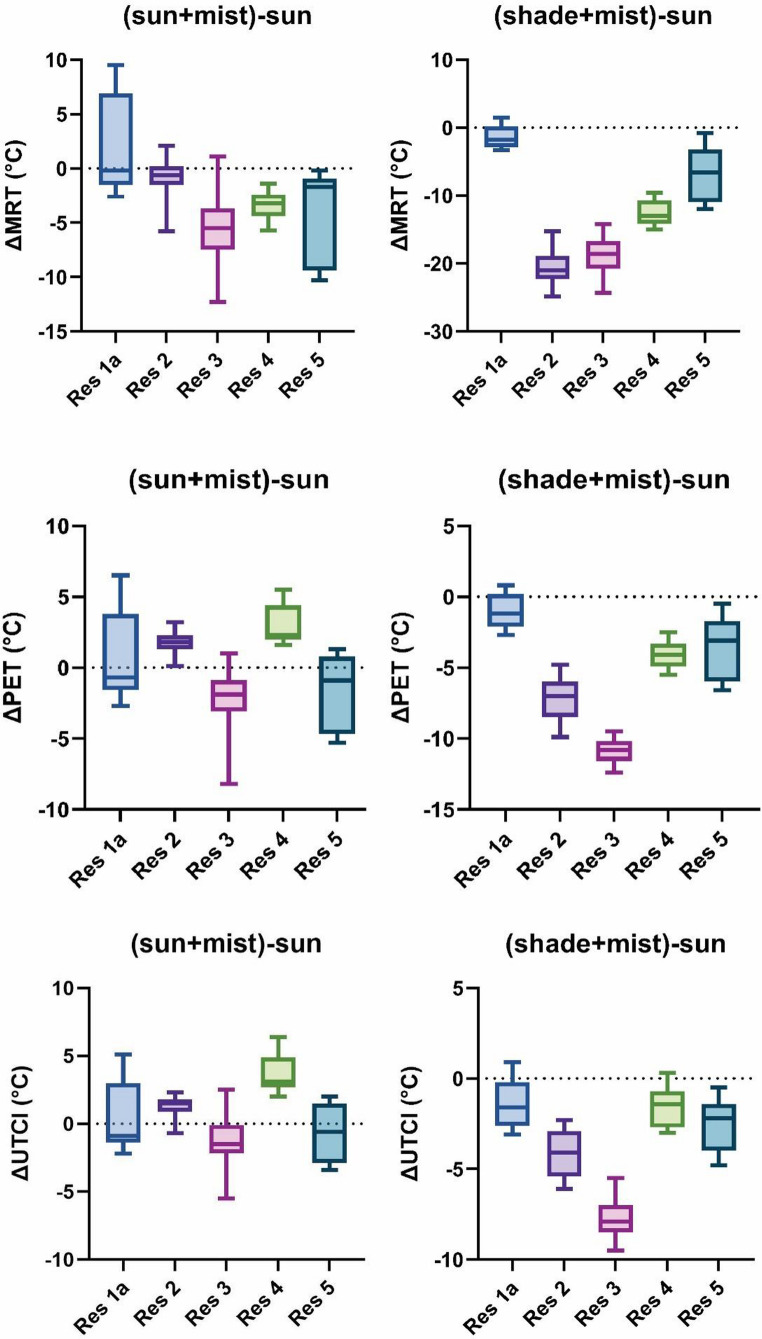
Boxplots of the differences of MRT (top), PET (middle), UTCI (bottom) between sun-only and the misted measurement spots (sun+mist; shade+mist) during the summer of 2022 at five restaurants in Novi Sad

Measurements in 2022 revealed distinct patterns in thermal comfort metrics across different sites. Analysis of mean values for MRT, UTCI, and PET across all measurement locations shows that shade+mist sites consistently demonstrate significantly lower values compared to sunny locations (Fig. S2–S4). On average, misting systems in shade locations reduced MRT by 12.0 °C, PET by 5.3 °C, and UTCI by 3.5 °C compared to sun-only locations, with maximum reductions (based on 10-minute averages) of 23.9 °C for MRT, 12.1 °C for PET, and 9.5 °C for UTCI being recorded (Fig. 1). When comparing sun+mist and shade+mist sites, shade+mist locations showed better performance, with MRT lower by 9.6 °C, PET by 5.6 °C, and UTCI by 4.2 °C on average, with maximum reductions of MRT by 22.4 °C, PET by 11.2 °C, and UTCI by 8.2 °C.

Comparisons between sunny locations showed significant variability in thermal comfort metrics, where sun-only locations compared to sun+mist locations showed an average increase in UTCI by 0.7 °C and in PET by 0.3 °C; however, in terms of MRT, an average reduction of 2.4 °C was observed for sun+mist locations. The thermal differentials between sunny locations demonstrated notable variability, ranging from − 9.5 °C to 12.3 °C for MRT, from − 6.5 °C to 6.1 °C for PET (with some outliers reaching 8.2 °C), and from − 6.4 °C to 5.2 °C for UTCI. These observed bidirectional variations in thermal comfort metrics indicate different responses to solar radiation and misting systems across measurement sites, and measurement equipment positioning (see Discussion).

Additionally, the comparative performance between sun-only and sun+mist locations showed location-specific variability. Measurement locations at Res3 and Res5 exhibited anticipated cooling patterns, with sun-only locations reporting higher average values than sun+mist locations across all calculated variables, confirming the effectiveness of misting systems in these specific environmental contexts. Conversely, at Res2 and Res4 locations, measurements in sun-only locations yielded lower PET and UTCI values than sun+mist locations (Fig. 1), contrary to the expected cooling effect of misting systems under solar exposure (see Discussion). The Res1a location, which reported the lowest values among all measurement sites, demonstrated lower averages across all calculated variables (MRT, PET, UTCI) in the sun-only site compared to the sun+mist site (Fig. S2–S4).

Analysis of the 2023 data also demonstrates substantial variability across measurement locations with varying environmental modifications. Fully exposed sunny locations consistently exhibited the highest thermal metric values, with sunny areas employing misting systems showing moderate improvement.

**Fig. 2:**
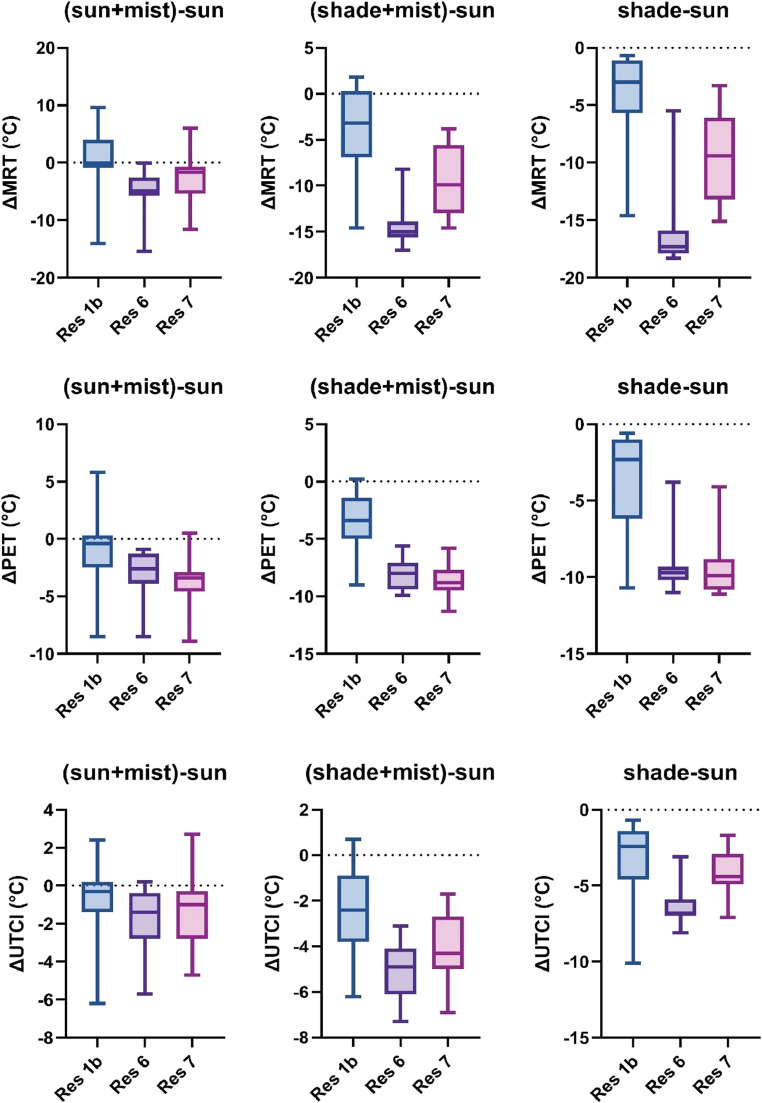
Boxplots of the differences of MRT (top), PET (middle), UTCI (bottom) between sun-only and the rest of the measurement spots (sun+mist; shade+mist; shade) during the summer of 2023 at three restaurants in Novi Sad

The cooling effect of misters in sun-exposed areas resulted in average decreases of approximately 2.9 °C for MRT, 2.7 °C for PET, and 1.3 °C for UTCI relative to sun-only locations (Fig. S5–S7). Peak cooling effects were substantially higher, with maximum reductions of 15.4 °C for MRT, 8.9 °C for PET, and 6.2 °C for UTCI being recorded (Fig. 2). A notable anomaly occurred at the Res1b location, where combined solar exposure and misting produced higher MRT values compared to the non-misted sun-exposed site (Fig. 2), suggesting possible local environmental interactions affecting radiative heat exchange processes. Consistent with 2022 data, the Res1 restaurant showed the lowest values across all calculated variables, attributable to its distinctive positioning.

#### Summer 2023

Analysis of the differential measurements between sunny and shaded locations revealed considerable variability (Fig. 2). The most notable thermal differences were observed between sun-only and shade-only locations, with an average reduction of MRT by 10.2 °C, PET by 7.5 °C, and UTCI by 4.6 °C, with variations ranging from 0.7 °C to 18.3 °C for MRT, 0.6 °C to 11.1 °C for PET, and 0.7 °C to 7.6 °C (10.1 °C outlier) for UTCI across measurement locations. The differences between sun-only locations and shade+mist locations were comparable to those at shade-only locations, with an average reduction of MRT by 9.4 °C, PET by 6.8 °C, and UTCI by 3.8 °C, ranging from − 1.8 °C to 17.0 °C for MRT, -0.2 °C to 11.3 °C for PET, and − 0.7 °C to 7.3 °C for UTCI.

Comparing shade+mist to shade-only locations, we observed differences in the calculated thermal comfort metrics, although these differences were less pronounced than in other comparisons. Contrary to expectations, misting systems in shaded environments demonstrated a slight increase across all measured variables. On average, misting systems increased MRT by 0.8 °C, PET by 0.7 °C, and UTCI by 0.8 °C compared to shade-only locations. Differences fluctuated from − 4.6 °C to 2.7 °C for MRT, from − 4.0 °C to 2.9 °C for PET, and from − 5.8 °C to 1.7 °C for UTCI. This finding underscores the complex interactions between passive and active cooling strategies in outdoor thermal comfort interventions that require further investigation.

#### Sensitivity analyses of meteorological features on thermal exposure

Sensitivity analyses of meteorological features on thermal exposure Regarding SHAP-based sensitivity analysis (see Fig. S8), under sun conditions, Tg exhibited the highest contribution to MRT (mean SHAP value = 9.1), while PET showed comparable sensitivity to Ta and Tg (SHAP values of 2.8 and 2.9, respectively). UTCI was primarily influenced by Ta (SHAP = 2.5), followed by Tg (SHAP = 2.1). For shade conditions, Tg remained the principal determinant of MRT (SHAP = 3.7), whereas Ta contributed most to PET and UTCI (SHAP values of 2.4 and 1.8, respectively). In the shade+mist condition, Tg exhibited the highest influence on MRT (SHAP = 4.2), while PET and UTCI were more sensitive to Ta (SHAP = 1.8 and 2.5, respectively), with RH and v showing minor but increased contributions to UTCI (SHAP = 0.8 and 0.3). In the sun+mist scenario, Tg exhibited the highest influence on MRT (SHAP = 7.0) and also contributed strongly to PET and UTCI (SHAP = 2.8 and 1.8), alongside notable contributions from Ta (SHAP = 2.9 for PET and 3.2 for UTCI). Across all conditions, Tg consistently dominated the prediction of MRT, while PET and UTCI exhibited sensitivity to Ta and Tg in a context-dependent manner. RH and v contributed relatively little overall, though their relevance increased marginally in misted environments, particularly for UTCI.

### Subjective thermal perception and evaluation of misting systems by restaurant guests

A total of 86 respondents (47 female and 39 male) completed the questionnaire during the observation period. Most respondents (57%) belonged to the 25–44 age group, followed by those aged 15–24 (21%). All respondents were sitting and were surveyed in the shade with mist.

**Fig. 3:**
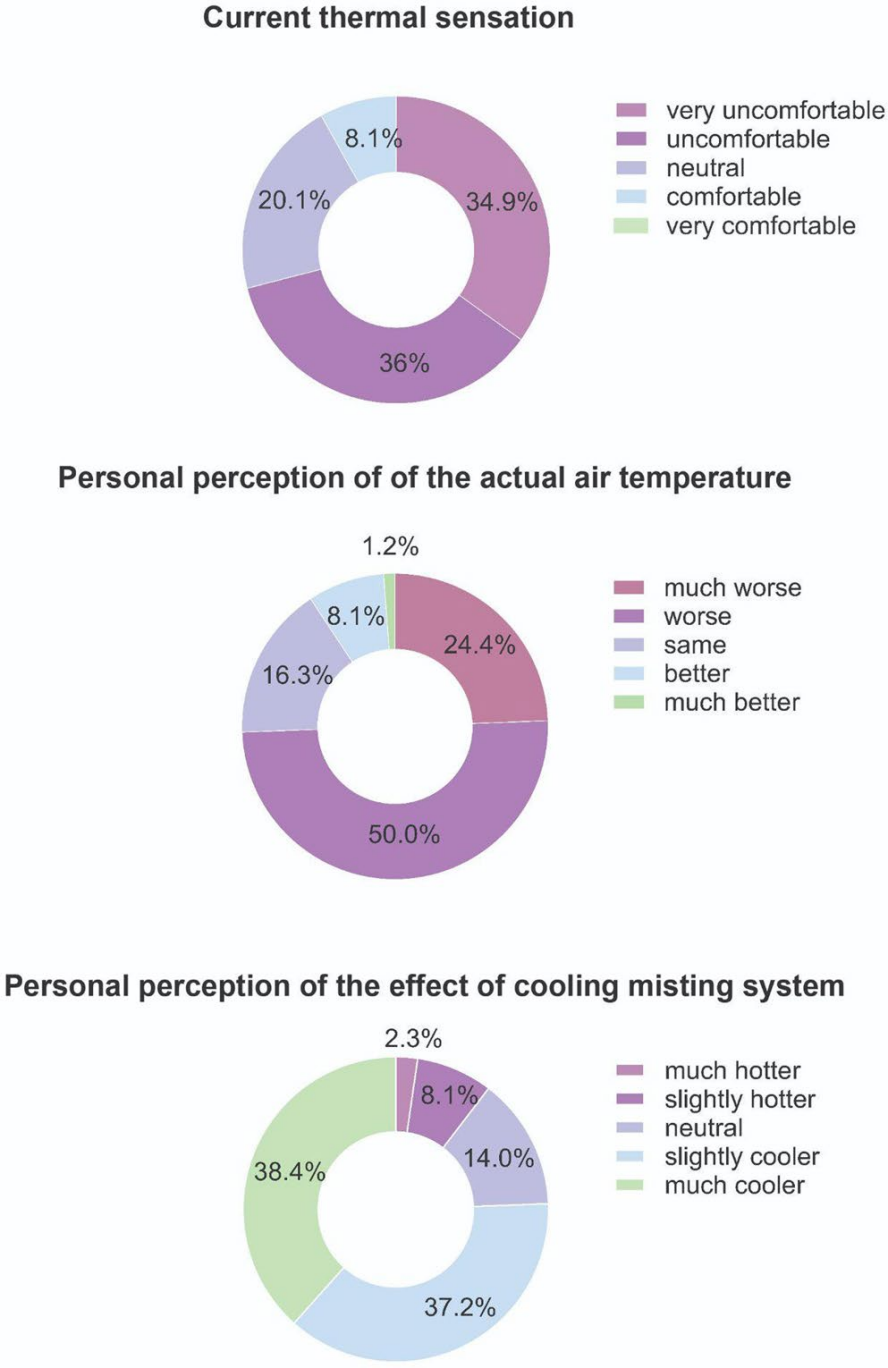
Perceived current thermal sensation (**a**), heat perception compared to the measured air temperature (**b**) and the perception of effect of cooling misting system (**c**)

When asked about their current thermal sensation, 71% of participants reported feeling uncomfortable or very uncomfortable (Fig. 3a), which corresponds to measured thermal indices indicating strong or extremely strong heat stress. The experienced thermal discomfort can partly explain why 74% of participants felt warmer than the measured air temperature, while 16% considered the thermal conditions to match the measured air temperature (Fig. 3b). Despite widespread discomfort and the perception of conditions warmer than the measured air temperature, 75% of respondents reported that misting made them feel cooler or much cooler, while 14% found no noticeable difference. Only 10% reported a negative impact (Fig. 3c) (see Discussion section).

## Discussion

### Discussion of the results

Despite limitations discussed below, the results presented in this study provide valuable insights into the effectiveness of evaporative cooling misting systems during hot summer days. Research gaps identified in the Introduction were addressed through measurements conducted under real-world conditions during restaurant operating hours in the temperate climate of a Central European city. To our knowledge, this is the first evaluation of this adaptation measure in restaurants in a temperate continental climate. The results are, however, considerably more variable than initially anticipated and different from those of studies conducted in other parts of the world.

The first campaign was conducted in summer 2022, including simultaneous measurements at three locations (sun+mist, shade+mist, and sun-only). Thermal indices show, consistent with Lehnert et al. (2021a); Vanos et al. (2022), that the most comfortable conditions were achieved at shade+mist seats, as expected (maximum reductions of 23.9 °C for MRT, 12.1 °C for PET, and 9.5 °C for UTCI). Similarly, shade+mist seats showed better thermal conditions compared to sun+mist seats (maximum reductions of 22.4 °C for MRT, 11.2 °C for PET, and 8.2 °C for UTCI). However, when comparing sun-only and sun+mist seats, substantial variability was found, ranging from − 9.5 °C to 12.3 °C for MRT, from − 6.5 °C to 6.1 °C for PET, and from − 6.4 °C to 5.2 °C for UTCI. These findings indicate location-specific variability. At some restaurants, the misting systems contributed to improved thermal comfort in sun-exposed locations as anticipated, while at others, they even worsened thermal conditions.

To ensure result consistency, the campaign was repeated the following summer (2023) with certain adjustments based on lessons learned during the first campaign. This time, we added one additional simultaneous measurement site (shade-only) to analyze the impact of misting systems in shaded locations. The results indicate that the reduction of thermal discomfort in sun-exposed sites with misting systems ranged from almost negligible to significant (maximum reductions of 15.4 °C for MRT, 8.9 °C for PET, and 6.2 °C for UTCI) compared to sun-exposed sites without evaporative misters. Comparisons between shade+mist and shade-only sites revealed that the misting systems in shade contributed to a slight increase across all thermal indices, indicating slightly increased discomfort. On average, misting systems increased MRT by 0.8 °C, PET by 0.7 °C, and UTCI by 0.8 °C compared to shade-only locations; however, skin temperature and other physiological responses to contact with cool mist were not investigated.

Focusing on the sun-exposed location, occasionally, higher MRT, UTCI, and PET values were found in sunny locations with misting systems (sun+mist) than at sunny locations without misting systems (sun-only). In these cases, sun+mist locations showed higher thermal exposure, not only for PET and UTCI, but also for MRT, and in some cases even higher air temperature. The results suggest variations in the evaporative cooling mister’s effectiveness among different measurement sites. Our findings indicate that although higher RH does influence PET and UTCI values at sunny locations with misting systems (sun+mist) compared to those without misting systems (sun), humidity is not the primary driver of this increased thermal exposure. Instead, the main factor appears to be the ratio between absorbed (heat that supports evaporation), reflected, and scattered radiation by water droplets (Tseng and Viskanta 2006) and the positioning of measurement equipment and misting systems in relation to the sun. We hypothesize that in our case, these radiative effects may outweigh any cooling benefit provided by evaporation. However, it should be noted that if we had also monitored skin temperature, the results could have been different due to skin cooling by conduction and evaporation occurring directly on the skin (Farnham et al. 2015; Oh et al. 2020). Therefore, further case studies in moderate climates with similar or more complex settings are needed. From a bioclimatological perspective, the observed thermal differences were most pronounced for MRT and least evident for UTCI, suggesting lower UTCI sensitivity in the context of this study. Similar patterns in Central Europe were reported by Jurek et al. (2025).

Further, the restaurants’ outdoor areas use different types of shading which may influence the performance of the evaporative misting systems as a cooling strategy. In our study, we encountered three types of shading: umbrellas (textile), awnings (textile, but with better coverage), and pergolas (solid material). Additionally, some of the restaurants are located near tall buildings which provide additional shade beyond the available shading types. For example, Res4 uses large textile umbrellas and is located in a square surrounded by the low-rise historical buildings in the city center. The same shading type is used at Res7. Middel et al. (2021) report that urban form shading types (e.g., buildings) reduce MRT most effectively, followed by trees and lightweight engineered structures (e.g., umbrellas). They also report that umbrella fabric radiates heat close to a person’s head, making them slightly less effective than shade from solid material (e.g., a bus station cover). According to Lee et al. (2018), individuals absorb more diffuse shortwave radiation under an umbrella than when shaded by a building. They report that in city centers, shade from buildings is more effective than umbrellas. Our results confirm that statement, given that Res1 (pergola + building shade) had significantly lower values in both measurement years. Similarly, Res6 benefits from the compound effect of a pergola (solid material) and building shade. The difference is that Res1 receives solar radiation in the early morning hours, while Res6 receives it during midday hours. Lee et al. (2018) found that the main determinant of shade efficacy was the shading strategy’s ability to prevent absorption of shortwave radiation, but other components of the human energy budget (such as sensible heat flux or longwave radiation absorption) should also be taken into account as they have a noticeable impact on shading strategy effectiveness.

The questionnaire survey shows more consistent results. Although thermal indices reveal variable performance of evaporative cooling systems, questionnaire results show that such interventions are well accepted by users of restaurant outdoor areas during hot summer days. The correlation between thermal perception assessment and actual temperature in the misting area is not very strong (Wang et al. 2024). Changes in thermal comfort after cooling were likely caused not only by changes in the physical environment (including skin temperature) but also by psychological factors (Farnham et al. 2015), such as thermal expectation. Several surveyed guests reported selecting specific restaurants due to the presence of misting systems. We can therefore expect a certain bias from thermal expectation (Nikolopoulou and Steemers 2003; Shooshtarian and Rajagopalan 2017; Květoňová et al. 2024), as respondents under misting systems expect that water will cool them down. Additionally, respondents under heat stress seek adaptive opportunities and tend to concentrate at seats under misting systems or inside air-conditioned restaurants, which can partially influence questionnaire results (Lehnert et al. 2021b). Furthermore, assessing cooling outcomes can be complicated when considering overall human thermal stress mitigation, since misters reduce human heat gain through decreased air temperature but impede evaporative heat dissipation through increased air humidity. Some studies suggest that increased humidity from mist spraying may exceed the human body’s tolerance limit, impacting subjective perception (Oh et al. 2020).

### Study limitations

Certain limitations should be noted. First, the two consecutive measurement campaigns included methodological differences that complicate direct comparisons. The 2022 measurement campaign used three measurement devices simultaneously per location, while the 2023 campaign used four devices. Additionally, the measurement time frame was shifted one hour earlier in the 2023 campaign. These adjustments were made based on lessons learned from the 2022 measurement campaign. Adding one additional measurement site (shade-only) enabled evaluation of the misters’ performance in shaded areas. The starting time was moved one hour earlier than in the previous year to avoid the need to move the instruments, because changes in the sun angle could potentially expose the shade measurement sites to sun and vice versa. Another reason is that according to the previous studies, Kestrel instruments tend to overestimate the air temperature (Sulzer et al. 2022), because the air temperature sensor is unshielded, so at lower sun angles, it could be directly exposed. In this context, only midday hours were included in the measurement campaign. The focus on midday hours was chosen to capture the hottest part of the day with the most stable meteorological conditions; however, including morning or evening hours might yield different results. These limitations also serve as recommendations for future research, which should spread to more restaurants and cities in temperate climate regions.

Nevertheless, the continuity of results at the Res1 site between 2022 and 2023—with consistent relationships among MRT, PET, and UTCI values across years—generally supports the validity of the measurement methodology. However, certain technical limitations exist in the use of a relatively small globe thermometer for calculating MRT. That said, globe thermometers are widely used in urban climate and bioclimatological research due to their practicality and affordability (Kántor and Unger 2011; Johansson et al. 2014; Lehnert et al. 2024). Using omnidirectional radiometers would be more accurate, particularly considering that water drops could impact the globe and influence measurements by lowering the globe temperature through both the cooling effect of water and latent heat loss from evaporation. Furthermore, the height of the misting systems, which slightly differs across the research locations, can affect the observed cooling performance, which is expected to decrease with the height of the misting system (Montazeri et al. 2017). Wind also affects performance; it can speed up water evaporation, and cause mist droplets to drift away from the target location. Non-directional wind could reduce the cooling efficiency of the misting system (Wang et al. 2024). The efficiency of this technology depends not only on its design, but also on the surrounding climatic context: wind speed and gusts dictate the severity of dilution (Ulpiani et al., 2019), while the water vapor pressure in the air determines the capacity to absorb additional moisture. More research under different wind speeds and water vapor pressure conditions could be valuable; however, in this study, the sensitivity analyses show rather minor direct effects of wind speed and humidity on MRT, PET, and UTCI.

Limitations also apply to the questionnaire survey for guests. The sample size was relatively small (*n* = 86), primarily due to low restaurant occupancy during the extreme meteorological conditions on measurement days. Furthermore, all guest respondents were surveyed in shade+mist, limiting the ability to compare thermal perceptions across different microclimatic conditions (sun, sun+mist, shade). The brief questionnaire, designed to minimize guest disruption, omitted potentially relevant variables including acclimatization status, clothing insulation, metabolic rates, and exposure duration (Lai et al. 2014). Additionally, the psychological effect of thermal expectation may have influenced responses, as the visible presence of misting systems may have created positive anticipation of cooling regardless of actual thermal conditions. Lehnert et al. (2021b) emphasized that infrastructure or facilities that provide adaptive opportunities particularly attract people under heat stress seeking cooling relief, leading to certain bias. Despite these limitations, the survey provides valuable insights into guest perceptions of misting systems. During fieldwork, restaurant managers informally reported that misting systems are generally perceived as beneficial for maintaining guest comfort and may positively influence customer attendance during hot weather conditions. However, visitor numbers were not formally monitored during the measurement campaigns, and no comparative attendance data were collected from restaurants without misting systems. It should also be noted that measurements were conducted during extremely hot midday periods, when overall occupancy was relatively low. In addition, detailed operational and economic data (e.g., installation costs, electricity and water consumption) were not systematically recorded, which limits a more comprehensive evaluation of the economic efficiency of misting systems.

## Conclusion

In this study, we evaluated the effectiveness of evaporative cooling misting systems in improving outdoor thermal comfort for users of restaurant outdoor seating areas. We achieved this by measuring meteorological parameters (T_a_, RH, v, and T_g_), calculating thermal indices (MRT, PET, UTCI), and conducting questionnaire surveys among restaurant guests to support the quantitative data. This enabled evaluation of both the objective effectiveness of misting systems based on measured values and the subjective user perceptions. Results demonstrate that misting systems in moderate climates are less effective as heat stress mitigation measures than in other climatic regions (e.g., hot arid). Findings confirmed that the most favorable thermal conditions, as expressed by MRT, UTCI, and PET, occur in shaded areas. Further research in moderate climates is needed to clarify the role of misting systems in sunny locations compared to sunny locations without misting systems, as our results were ambiguous. Nevertheless, based on questionnaire results, respondents sitting under misting systems consistently reported improved thermal comfort, likely due to direct skin cooling from water droplets and/or psychological factors related to thermal expectation. The correlation between thermal perception and measured thermal exposure in misted areas is not strong, and this discrepancy warrants further attention from researchers. These findings provide practical insights for the design and operation of misting systems in private outdoor hospitality spaces, underscoring the importance of shading conditions and the spatial configuration of outdoor environments in enhancing thermal comfort during hot weather conditions. For future research, we recommend investigating morning and evening hours, spring and autumn seasons, and optimal misting types and intervals for maximum efficiency.

The findings of this study have broader implications for urban well-being, sustainable urban development, and climate change adaptation. By providing evidence on the effectiveness and limitations of evaporative cooling misting systems as a heat stress measure, this study contributes to Sustainable Development Goal 3 (SDG 3) through improving thermal comfort and well-being of urban populations of all ages during extreme heat events. The evidence-based insights into the performance of cooling interventions in outdoor hospitality settings support SDG 11 by informing urban planners and local authorities about viable adaptation measures for climate-resilient cities, including those in temperate climates. Finally, by expanding the evidence base for cost-effective urban cooling strategies, this study contributes to SDG 13 in the context of increasing frequency and intensity of heat waves.

## Supplementary Information

Below is the link to the electronic supplementary material.

Supplementary Material 1 (DOCX 635 KB)

## Data Availability

The data are available from the corresponding author [V.K.].
