# Supplementary material for: Cooling effect of evaporative misters in outdoor restaurant areas: case study of Novi Sad, Serbia
Source: Int J Biometeorol. 2026 Apr 24;70(5):139. doi: 10.1007/s00484-026-03203-7 (PMC13109214; doi:10.1007/s00484-026-03203-7)
Supplement: Supplementary file 1 — (DOCX 635 KB) [file 484_2026_3203_MOESM1_ESM.docx]

**Supplementary material – Cooling effect of evaporative misters in outdoor restaurant areas: Case study of Novi Sad, Serbia**

**Tab. S1. Site specific characteristics of the restaurant outdoor seating areas in Novi Sad**

| Restaurant | Year(s) of measurement | LCZ | Site orientation | Misting type | Misting mode | Shading type | Shading height |
| --- | --- | --- | --- | --- | --- | --- | --- |
| Res1 (a+b) | 2022+2023 | LCZ 3_2,_ canyon | NE | Sprinklers and fans | 20s On- 20s off, oscillating fans | Pergola – solid material + shade from the building | 2.5-3 m |
| Res2 | 2022 | LCZ 5 | NE | Sprinklers | On-off | Awning (textile) | 2.5-3 m |
| Res3 | 2022 | LCZ 3 | NE | Sprinklers | On-off | Awning (textile) | 2.5-3 m |
| Res4 | 2022 | LCZ 3 | NW | Fans | On-off, oscillating | Umbrella (textile) | 2.5-3 m |
| Res5 | 2022 | LCZ 5 | SW | Sprinklers | On-off | Awning (textile) | 2.5-3 m |
| Res6 | 2023 | LCZ 2 | SW | Sprinklers | 20s On- 30s off | Pergola – solid material + shade from the building | 2.5-3 m |
| Res7 | 2023 | LCZ 3 | SE | Sprinklers | 10s On- 10s off | Umbrella (textile) | 2.5-3 m |

**Tab. S2**. Accuracy, resolution and range of Kestrel 5400 Heat Stress Tracker sensors

| **Sensor** | **Accuracy (+/-)** | **Resolution** | **Range** |
| --- | --- | --- | --- |
| Air temperature | 0.5 ºC | 0.1 ºC | -29.0 to 70.0 ºC |
| Relative humidity | ±2% RH | 0.1 % RH | 10 to 90 % 25 ºC non-condensing |
| Wind speed | Larger than 3% of reading, least significant digit of 20 ft/min | 0.1 m/s | 0.6 to 40.0 m/s |
| Globe temperature | 1.4 ºC | 0.1 ºC | -29.0 to 60.0 ºC |

**Tab. S3.** Background data on Ta and RH on the measurement days

| Measurement day | Ta_max_ | Ta_min_ | Ta_14h_ | RH_14h_ |
| --- | --- | --- | --- | --- |
| 04/07/2022 | 36.2°C | 21.0°C | 35.8°C | 23% |
| 18/08/2022 | 38.0°C | 19.3°C | 36.8°C | 29% |
| 19/08/2022 | 39.2°C | 20.9°C | 39.2°C | 26% |
| 15/07/2023 | 32.8°C | 18.1°C | 31.4°C | 46% |
| 16/07/2023 | 36.2°C | 19.5°C | 35.8°C | 48% |
| 17/07/2023 | 37.2°C | 22.4°C | 36.2°C | 45% |

Source: Republic Hydrometeorological Service

**Tab. S4**. PET and UTCI categories and index threshold values for the stress level of humans (according to Jendritzky et al. [1990], Matzarakis et al. [1999], and Błażejczyk et al. [2014]).

| PET stress level | PET [°C] | UTCI stress level | UTCI [°C] |
| --- | --- | --- | --- |
| Strong heat stress | 35.1–41.0 | Very strong heat stress | 38.1–46.0 |
| Moderate heat stress | 29.1–35.0 | Strong heat stress | 32.1–38.0 |
| Slight heat stress | 23.1–29.0 | Moderate heat stress | 26.1–32.0 |
| No thermal stress | 18.1–23.0 | No thermal stress | 9.1–26.0 |
| Slight cold stress | 13.1–18.0 | Slight cold stress | 0.1–9.0 |
| Moderate cold stress | 8.1–13.0 | Moderate cold stress | -12.9 – 0 |
| Strong cold stress | 4.1–8.0 | Strong cold stress | -26.9 – -13.0 |
| Extreme cold stress | below 4.0 | Very strong cold stress | - 39.9 – -27.0 |
|  | | Extreme cold stress | below -40.0 |

**Fig. S1**. Questionnaires for Restaurant Guests (translated from Serbian)

**Questionnaire for Restaurant Guests**

Thermal comfort measurements are being conducted by the Urban Climate Research Group at the Faculty of Sciences, University of Novi Sad. We kindly ask you to complete a short questionnaire about your subjective thermal sensation while sitting in the restaurant’s outdoor area. The questionnaires are anonymous, and the data will be used exclusively for scientific purposes.

1. Gender M F
2. Age group 15-24 25-44 45-64 65+
3. My current subjective thermal sensation is: *(please mark X next to the appropriate smiley)*

very uncomfortable uncomfortable neutral comfortable very comfortable


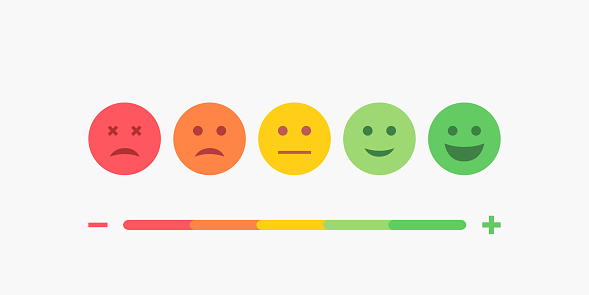


1. My subjective heat perception is better or worse compared to the measured air temperature

much worse worse same better much better


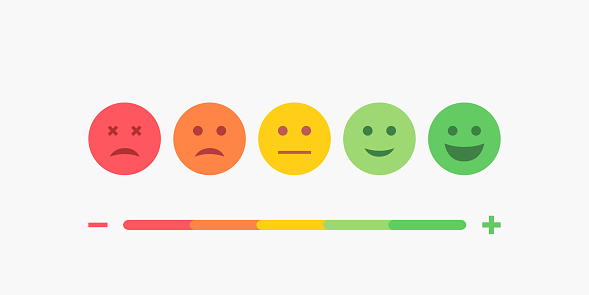


1. My thermal perception when sitting in the restaurants outdoor area with the evaporative cooling misters?

much hotter slightly hotter neutral slightly cooler much cooler


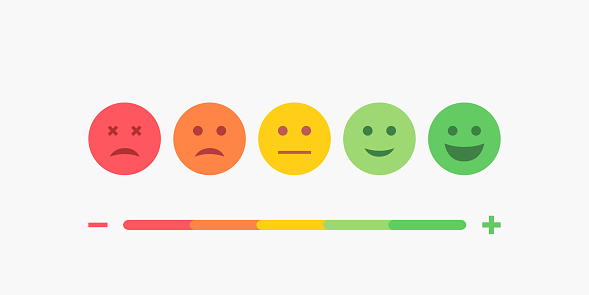


Thank you for the cooperation!


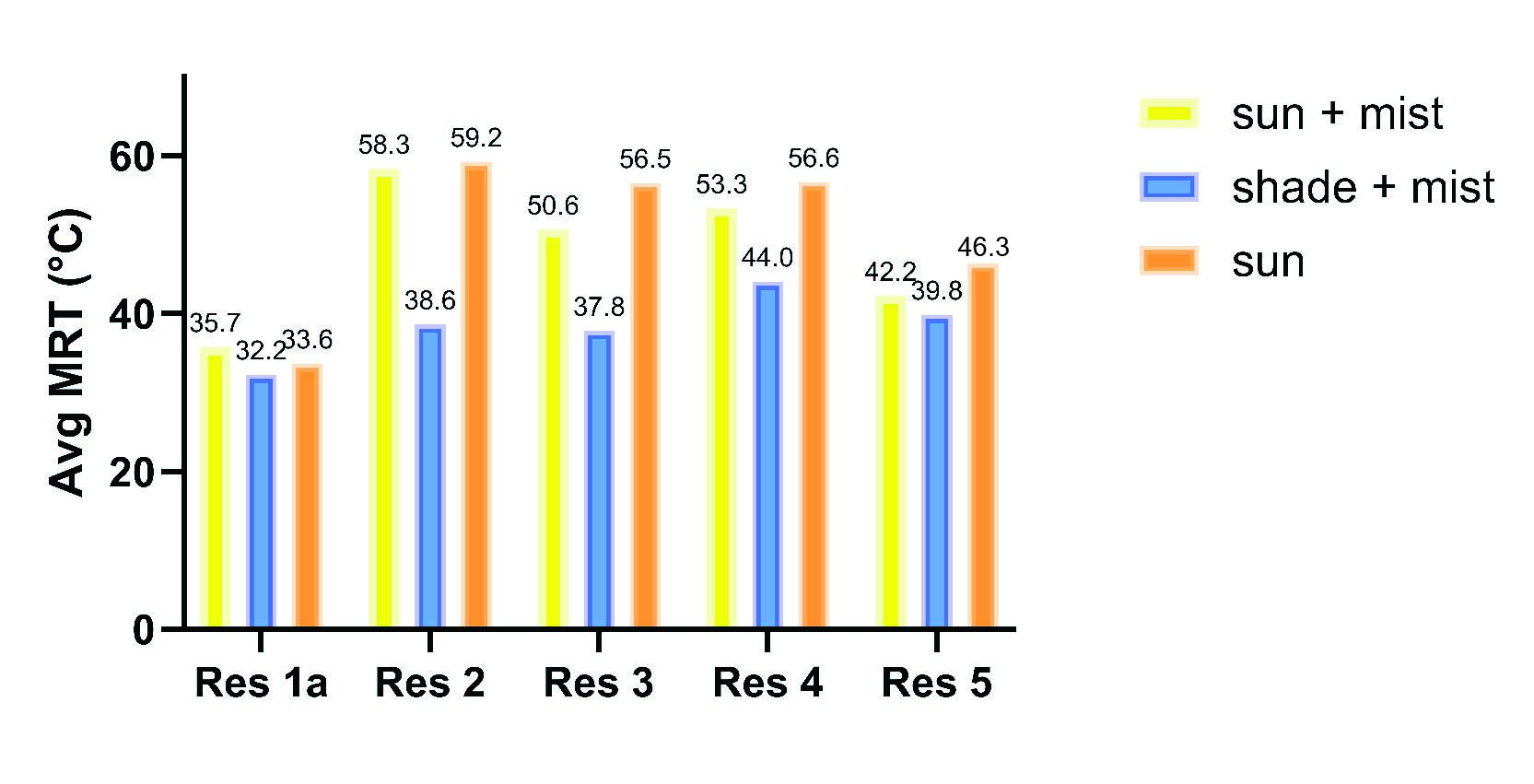


**Fig. S2**. Average MRT at three measurement spots (sun+mist; shade+mist; sun) during the summer of 2022 at five restaurants in Novi Sad.


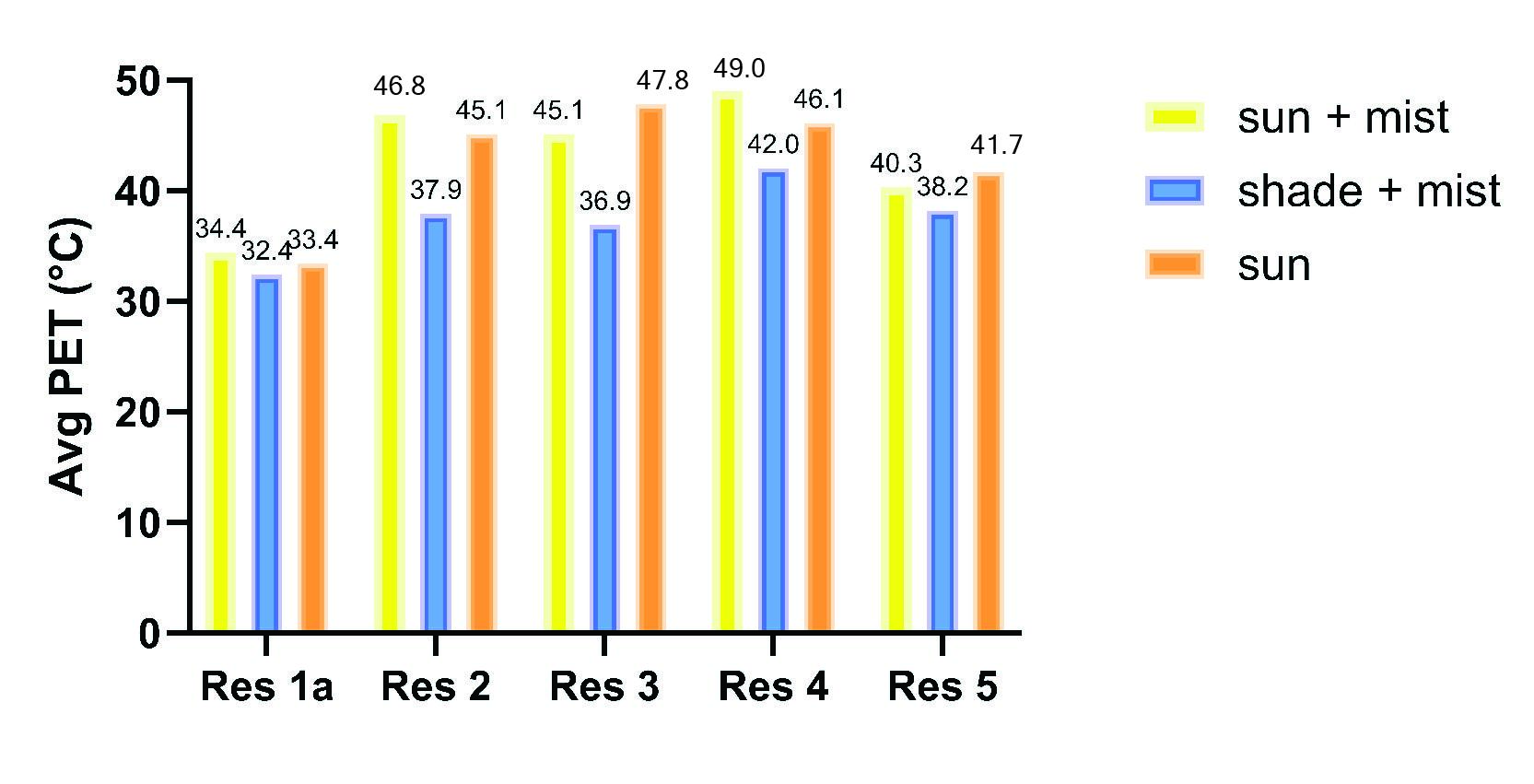


**Fig. S3**. Average PET at three measurement spots (sun+mist; shade+mist; sun) during the summer of 2022 at five restaurants in Novi Sad.


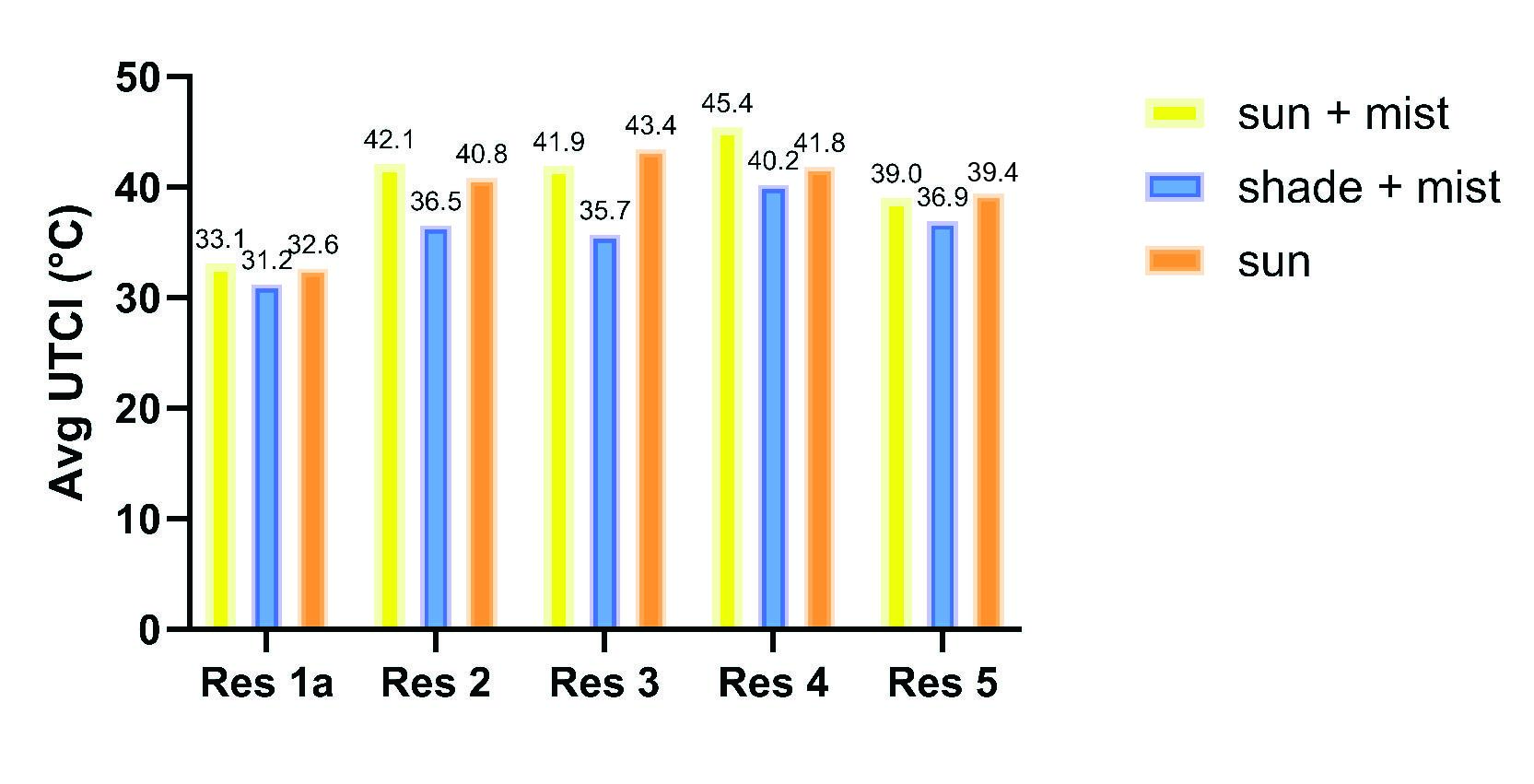


**Fig. S4**. Average UTCI at three measurement spots (sun+mist; shade+mist; sun) during the summer of 2022 at five restaurants in Novi Sad.


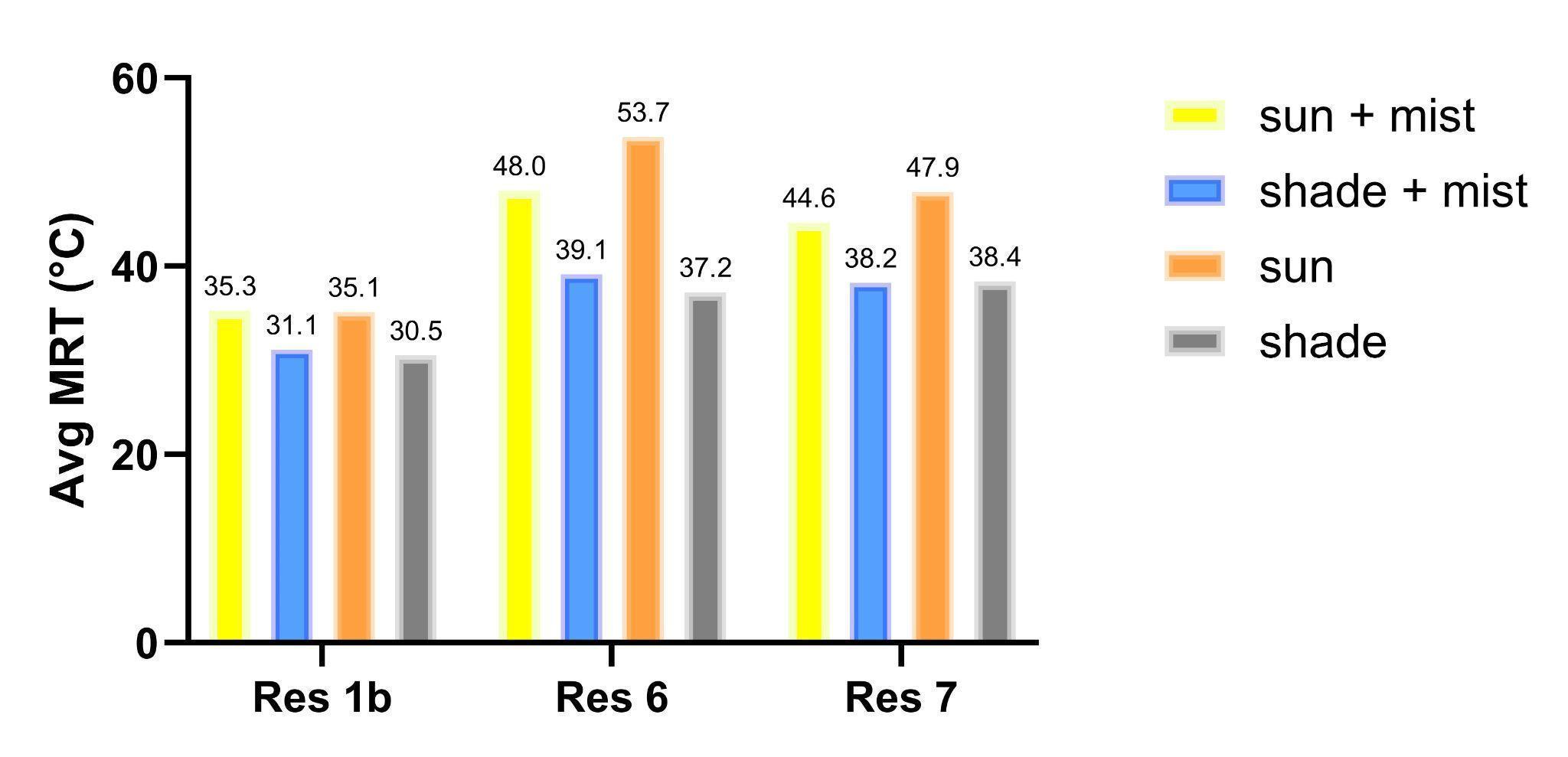


**Fig. S5**. Average MRT at four measurement spots (sun+mist; shade+mist; sun; shade) during the summer of 2023 at three restaurants in Novi Sad.


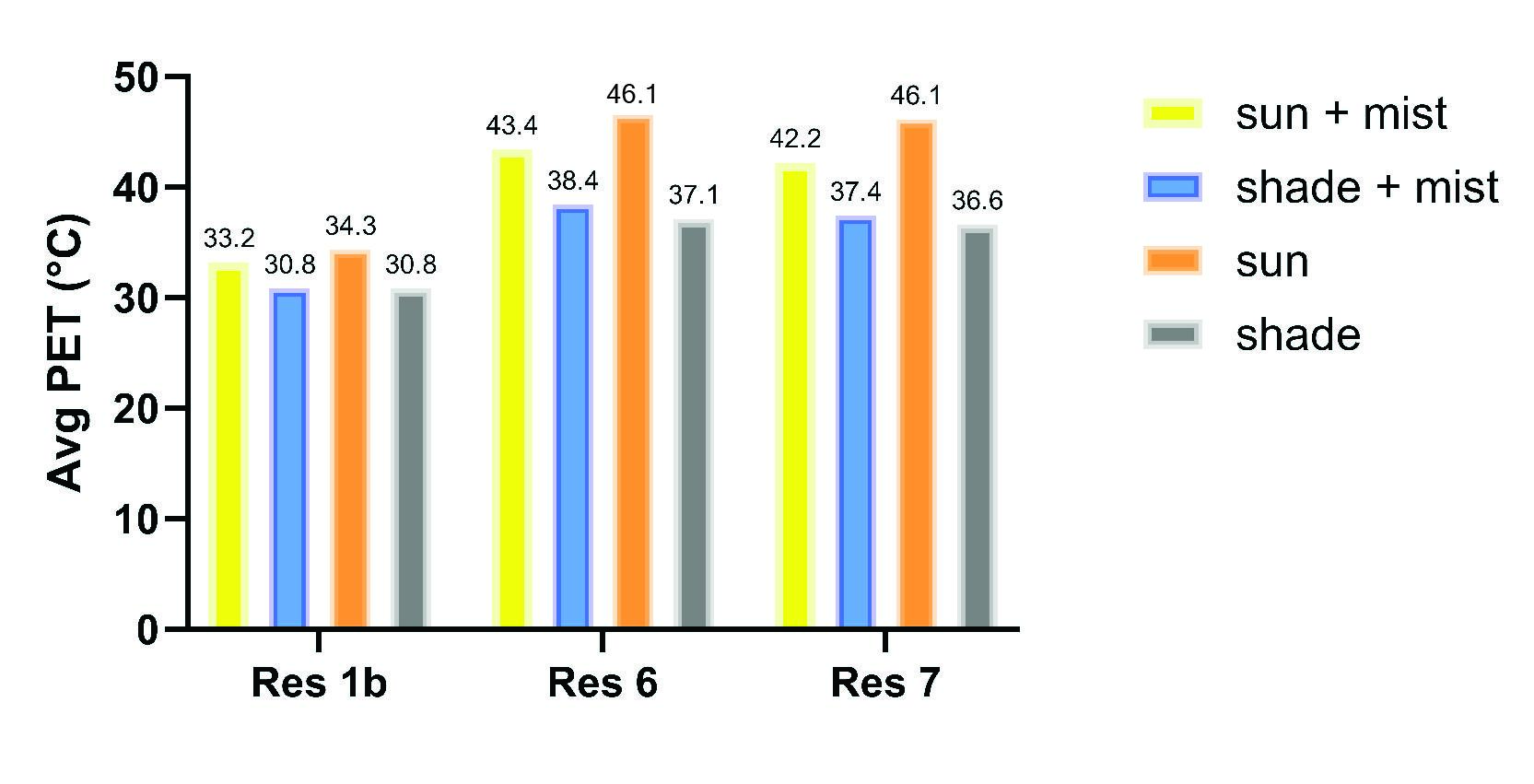


**Fig. S6**. Average PET at four measurement spots (sun+mist; shade+mist; sun; shade) during the summer of 2023 at three restaurants in Novi Sad.


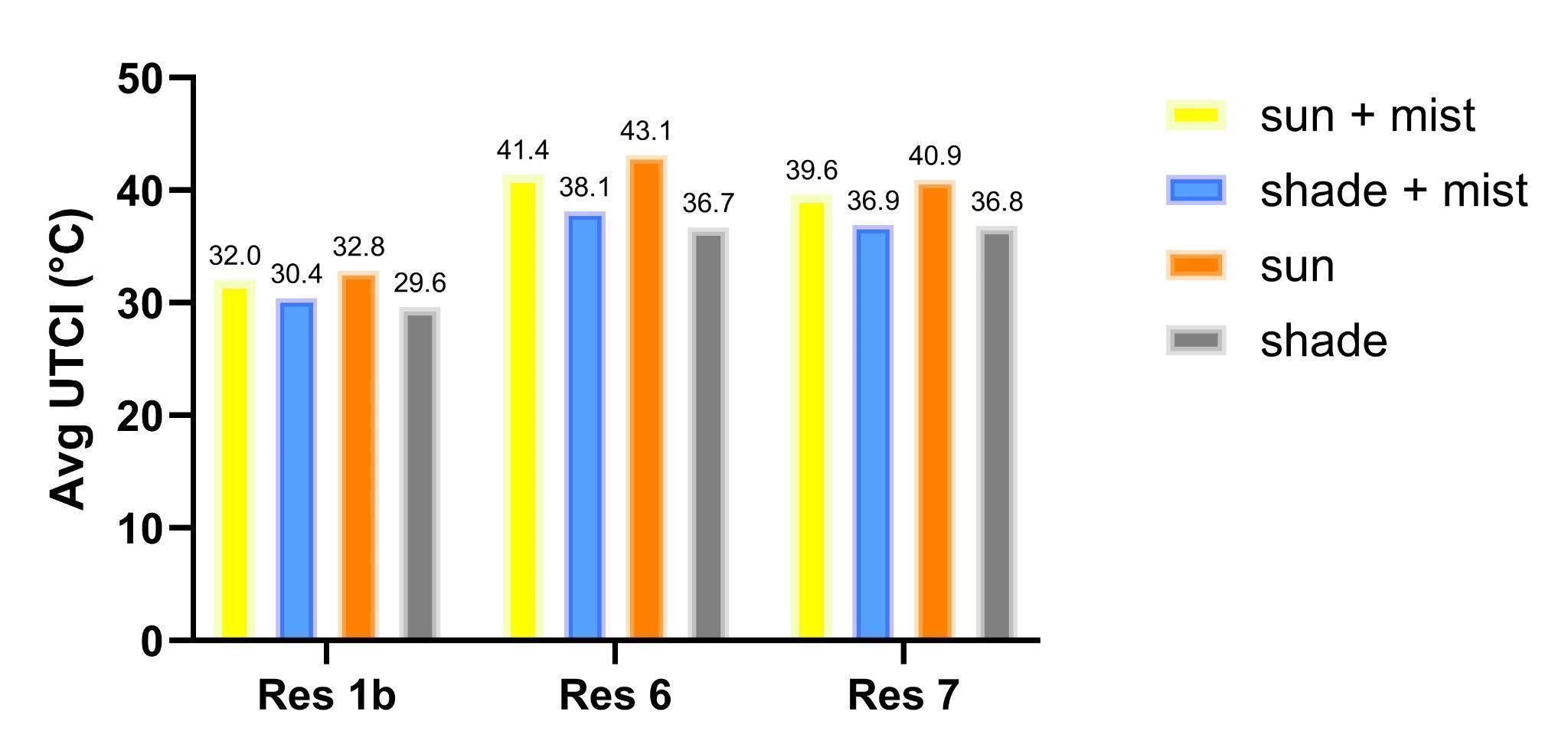


**Fig. S7**. Average UTCI at four measurement spots (sun+mist; shade+mist; sun; shade) during the summer of 2023 at three restaurants in Novi Sad.


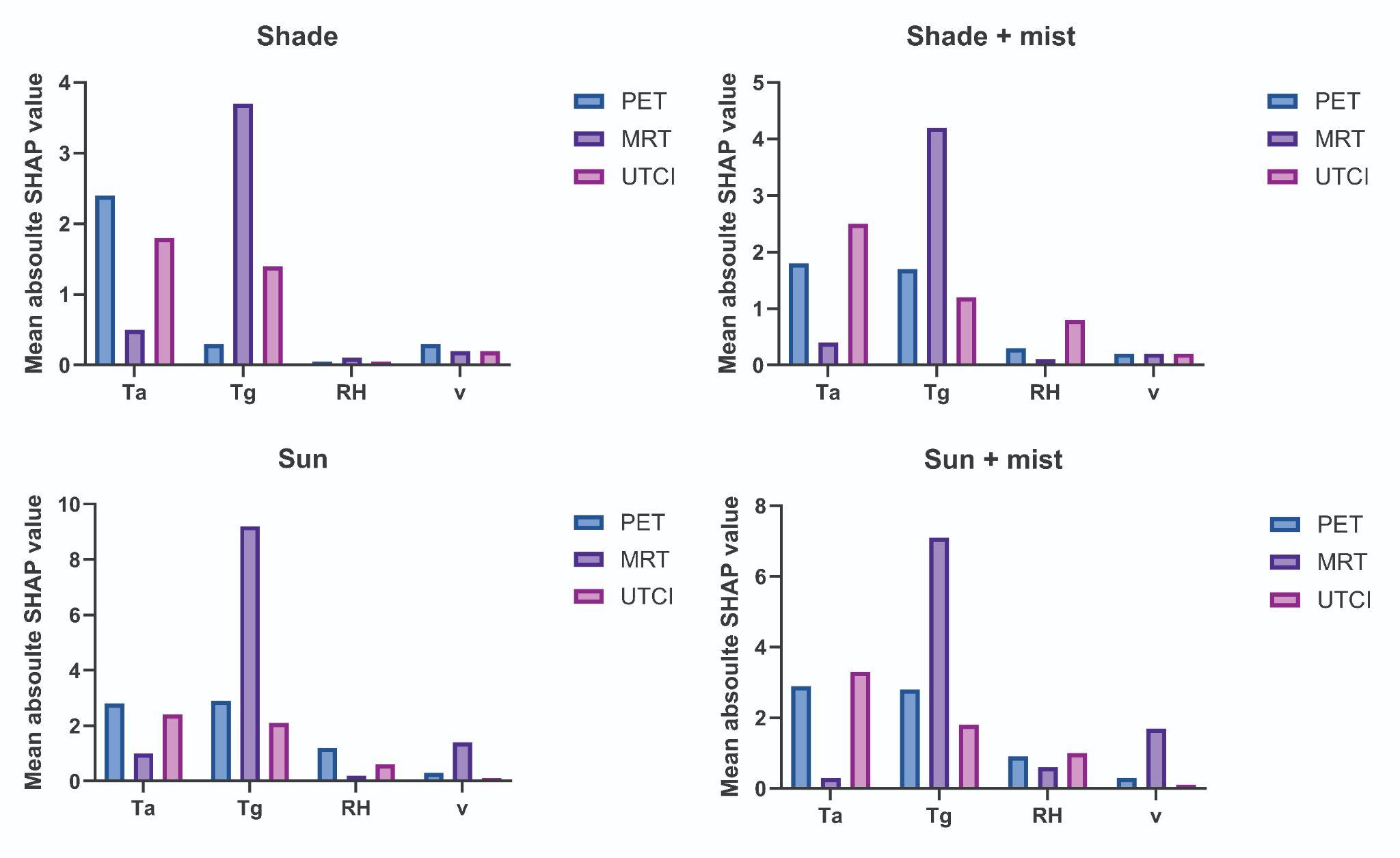


**Fig. S8**. SHAP feature importance analysis for PET, MRT, UTCI across different measurement conditions (sun; sun+mist; shade+mist; shade)
